# Astragaloside IV ameliorates renal injury in db/db mice

**DOI:** 10.1038/srep32545

**Published:** 2016-09-02

**Authors:** Huili Sun, Wenjing Wang, Pengxun Han, Mumin Shao, Gaofeng Song, Heng Du, Tiegang Yi, Shunmin Li

**Affiliations:** 1 Department of Nephrology, Shenzhen Affiliated Hospital, Guangzhou University of Chinese Medicine, Shenzhen, China; 2 Department of Pathology, Shenzhen Affiliated Hospital, Guangzhou University of Chinese Medicine, Shenzhen, China; 3 Department of Biological Sciences, the University of Texas at Dallas, Richardson, Texas, USA

## Abstract

Diabetic nephropathy is a lethal complication of diabetes mellitus and a major type of chronic kidney disease. Dysregulation of the Akt pathway and its downstream cascades, including mTOR, NFκB, and Erk1/2, play a critical role in the development of diabetic nephropathy. Astragaloside IV is a major component of Huangqi and exerts renal protection in a mouse model of type 1 diabetes. The current study was undertaken to investigate the protective effects of diet supplementation of AS-IV on renal injury in db/db mice, a type 2 diabetic mouse model. Results showed that administration of AS-IV reduced albuminuria, ameliorated changes in the glomerular and tubular pathology, and decreased urinary NAG, NGAL, and TGF-β1 in db/db mice. AS-IV also attenuated the diabetes-related activation of Akt/mTOR, NFκB, and Erk1/2 signaling pathways without causing any detectable hepatotoxicity. Collectively, these findings showed AS-IV to be beneficial to type 2 diabetic nephropathy, which might be associated with the inhibition of Akt/mTOR, NFκB and Erk1/2 signaling pathways.

Early diabetic nephropathy (DN) is characterized by hyperfiltration and microalbuminuria. Glomerular basement membrane (GBM) and tubular basement membrane (TBM) thickening and mesangial expansion are featured pathologies of DN and develop as the disease progresses, eventually leading to renal failure^1^. However, the molecular mechanism underlying DN is still largely undetermined, and the effective approaches for the treatment of DN are extremely insufficient^2^. Akt, also known as protein kinase B, and its downstream cascades, including mammalian target of rapamycin (mTOR), nuclear factor kappa B (NFκB), and extracellular signal-regulated kinase 1 and 2 (Erk1/2) play a critical role in cell survival and proliferation^3^. Dysregulation of Akt pathway is implicated in many human diseases including cancer, diabetes, and cardiovascular and neurological diseases^4^. Recent studies have suggested that the activation of Akt signaling pathway is closely associated with the development of DN^5^,^6^,^7^. Inhibition of Akt, its downstream cascades, or both by using statin, troglitazone, sirolimus, or rapamycin has a protective effect on DN^8^,^9^,^10^,^11^.

Astragaloside IV (AS-IV) is the major component of Huangqi (*Radix Astragali Mongolici*) which is the dried root of the leguminous plants *Mongolia Astragalus* [*Astragalus membranaceus (Fisch.) Bge.*]. The main components in Huangqi are polysaccharides, saponins and flavonoids. AS-IV has been identified as one of the main active compounds of the saponins. The molecular formula for AS-IV is C_41_H_68_O_14_ and its molecular weight is 784.97. AS-IV has anti-inflammation, anti-viral, and anti-cancer effects^12^. It is used for the treatment of a variety of diseases including cardiac^13^, hepatic^14^,^15^,^16^ and neurological disorders^17^,^18^,^19^. The protective effect of AS-IV has been mechanically linked to its inhibition of Akt and its downstream pathways^20^,^21^,^22^. Notably, recent studies have shown that AS-IV preserves renal function in a mouse model of type 1 diabetes^23^,^24^. However, whether AS-IV protects renal function in an *in vivo* setting mimicking type 2 diabetes has not previously been investigated.

In this study, the influence of diet supplementation of AS-IV on DN was examined in db/db mice, a type 2 diabetic mouse model. The status of Akt and its downstream cascades were here investigated in diabetic mice treated with AS-IV.

## Results

### AS-IV reduces albuminuria without suppressing diabetic glomerular hyperfiltration

**Figure 1:**
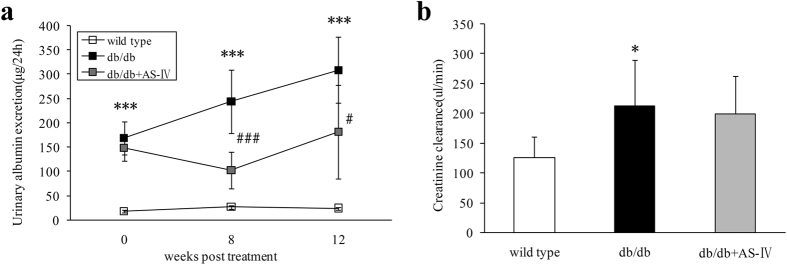
Urinary albumin excretion (μg/24h) after 8 weeks and 12 weeks treatment in each group (n = 6–8). The db/db mice showed significantly more urinary albumin excretion than wild type, and AS-IV therapy was found to ameliorate this to a considerable extent (Fig. 1a). Creatinine clearance (μl/min) calculated at 12 weeks indicated an obvious increase in db/db mice, but AS-IV treatment was not found to reduce it (Fig. 1b). *P < 0.05 and ***P < 0.001 when compared to wild type. ^#^*P* < 0.05 and ^###^*P* < 0.001 when compared to the db/db group.

Compared to wild type mice, albuminuria was established in diabetic db/db mice at the initial treatment and gradually increased (Fig. 1a). At the end of study, db/db mice showed elevated creatinine clearance rate (Ccr) which indicated significant glomerular hyperfiltration (Fig. 1b). After 8 weeks of AS-IV treatment, albuminuria had decreased significantly and the effect continued through 12 weeks (Fig. 1a). However, 12 weeks of AS-IV treatment did not suppress diabetic glomerular hyperfiltration (Fig. 1b).

### Changes in physiological and metabolic parameters

**Figure 2:**
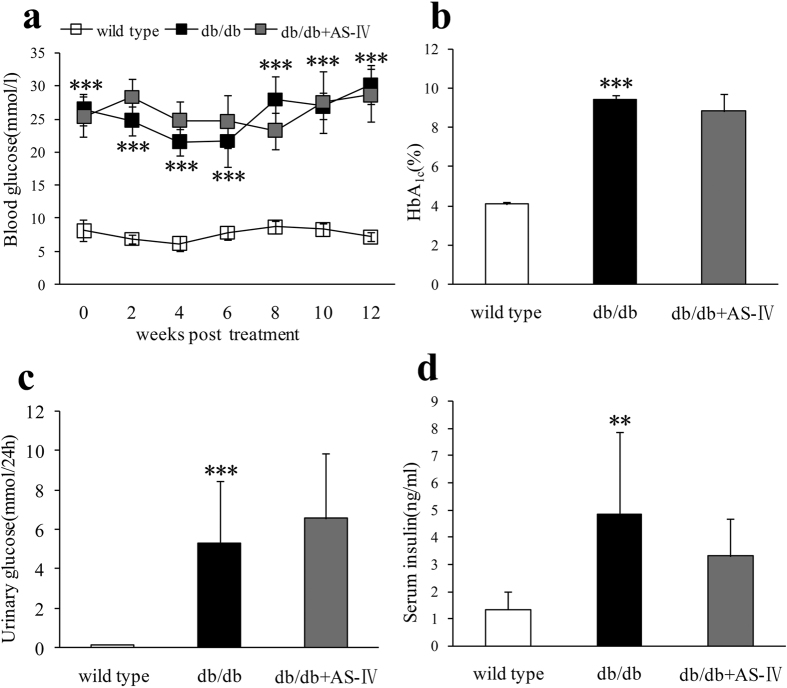
The db/db group displayed higher blood glucose than the wild type group throughout the experiment and higher glycated HbA1c, urinary glucose, and serum insulin at 12 weeks. There was no significant difference in these indexes after AS-IV therapy (Fig. 2a–d). n = 8 per group. **P < 0.01 and ***P < 0.001 when compared to wild type group.

**Figure 3:**
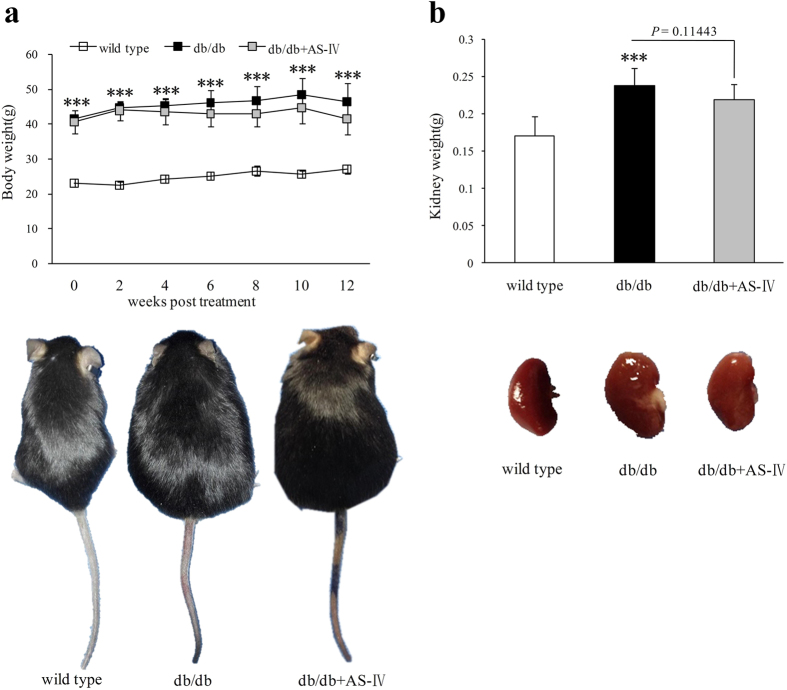
Throughout the experiment, db/db mice manifested heavier body weight than wild type and, at the end of the study, they showed heavier kidneys (Fig. 3a,b). In mice treated with AS-IV, body weight and kidney weight both showed declining trends but not to significant degree (Fig. 3a,b). n = 8 per group. ***P < 0.001 when compared to wild type group.

Unlike wild type mice, db/db mice displayed polydipsia and polyuria from their initial treatment and increased feces production starting at 8 weeks. AS-IV therapy improved these symptoms obviously (Table 1). Untreated db/db mice showed higher blood glucose during the experiment period which was consistent with elevated HbA_1C_ at the end of study (Fig. 2a,b). Urinary glucose and serum insulin in db/db mice at week 12 also increased obviously (Fig. 2c,d). However, there were no significant differences in these parameters after AS-IV treatment (Fig. 2a–d). During the experiment, db/db group mice manifested heavier body weight from initial therapy and heavier kidney weight at the end of the study. The body weight of mice in the AS-IV treatment group showed a declining trend after 4 weeks of therapy. At 12 weeks, the kidney weight also declined but not to a significant extent (Fig. 3a,b).

### AS-IV reduces glomerular hypertrophy and injury

**Figure 4:**
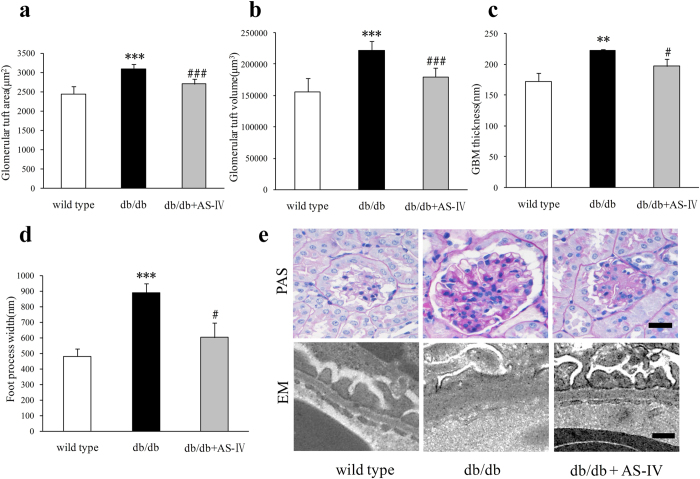
At 12 weeks, db/db mice displayed larger GTA and GTV (n = 6 per group), thicker GBM and wider FPW (n = 3 per group) relative to wild type mice. AS-IV treatment reversed these alterations approach to normal condition (Fig. 4a–d). PAS staining and EM images were used to depict these characteristics in each group (Fig. 4e). Scale bars, 20 μm for PAS images, 200 nm for EM. **P < 0.01 and ***P < 0.001 relative to wild type group. ^#^*P* < 0.05 and ^###^*P* < 0.001 relative to the db/db group.

**Figure 5:**
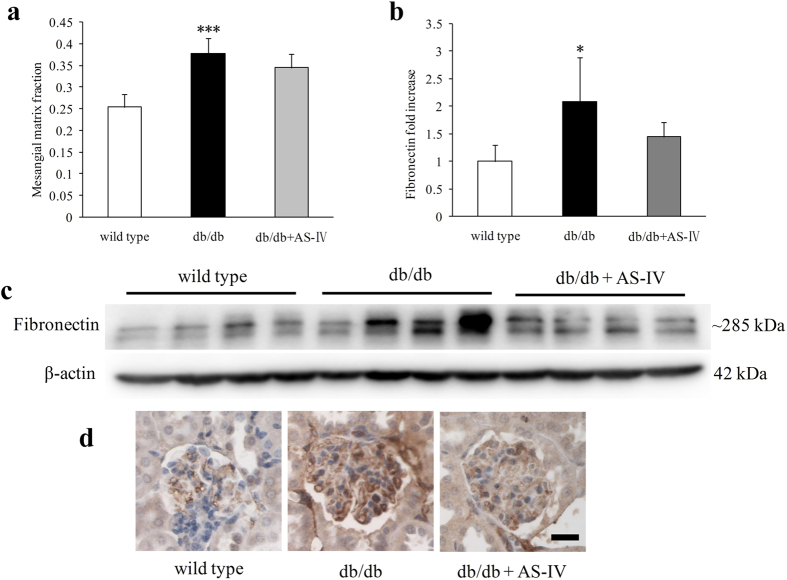
The mesangial matrix fraction (n = 6 per group) was significantly higher in db/db mice (Fig. 5a). Fibronectin content in the renal cortex was also visibly higher, as indicated by Western blot analysis (n = 4 per group) and immunohistochemistry (Fig. 5b–d). AS-IV treatment was found to reduce mesangial matrix fraction and fibronectin level but not to a significant degree (Fig. 5a,b). *P < 0.05 and ***P < 0.001 relative to the wild type group.

At 12 weeks, db/db mice displayed larger glomerular tuft area (GTA), greater glomerular tuft volume (GTV), thicker GBM, and wider foot process width (FPW) than wild type mice. AS-IV treatment reversed these alterations approach under normal conditions (Fig. 4a–d). Periodic acid-Schiff (PAS) staining and electron microscopy (EM) were used to assess these characteristics in each group (Fig. 4e). In db/db mice, the mesangial matrix fraction increased significantly (Fig. 5a). Fibronectin content in the renal cortex also increased visibly, as indicated by Western blot and immunohistochemistry (Fig. 5b–d). AS-IV treatment was found to reduce mesangial matrix fraction and the level of fibronectin but not to a significant degree (Fig. 5a,b).

### AS-IV ameliorates tubular injury

**Figure 6:**
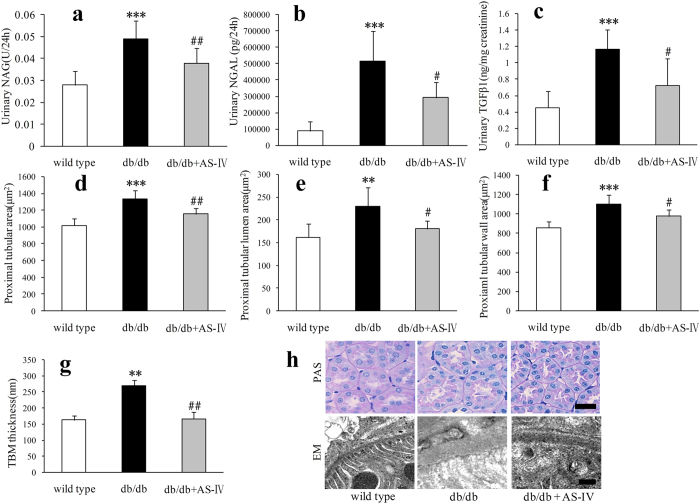
In db/db mice, urinary NAG, NGAL and TGF-β1 excretion increased significantly (Fig. 6a–c). n = 6–10 per group. The proximal tubular area, lumen and wall (n = 6 per group) became larger and TBM (n = 3 per group) became thicker than in wild type mice (Fig. 6d–h). Figure 6a–c showed AS-IV treatment significantly reduce urinary NAG, NGAL and TGF-β1. The proximal tubular area, lumen, and wall became smaller (Fig. 6d–f). The TBM also became thinner than db/db mice (Fig. 6g). The representative images of tubular PAS staining and TBM are shown in Fig. 6h. Scale bars, 20 μm for PAS images, 200 nm for EM. **P < 0.01 and ***P < 0.001 relative to the wild type group. ^#^*P* < 0.05 and ^##^*P* < 0.01 relative to the db/db group.

In db/db mice, urinary N-acetyl-β-d-glucosamine-dase (NAG), neutrophil gelatinase-associated lipocalin (NGAL) and TGF-β1 excretion was found to have increased significantly at 12 weeks (Fig. 6a–c). The proximal tubular area, lumen and wall became larger and TBM became thicker than in wild type mice (Fig. 6d–h). AS-IV treatment significantly reduced urinary NAG, NGAL and TGF-β1 (Fig. 6a–c). The proximal tubular area, lumen, and wall were reduced to nearly normal conditions (Fig. 6d–f). The TBM also became thinner than in db/db mice (Fig. 6g,h).

### AS-IV suppresses activation of p-Akt(Ser473), p-mTOR(Ser2448), p-NF-κB p65(Ser536) and p-Erk1/2(Thr202/Tyr204) as assessed at 12 weeks

**Figure 7:**
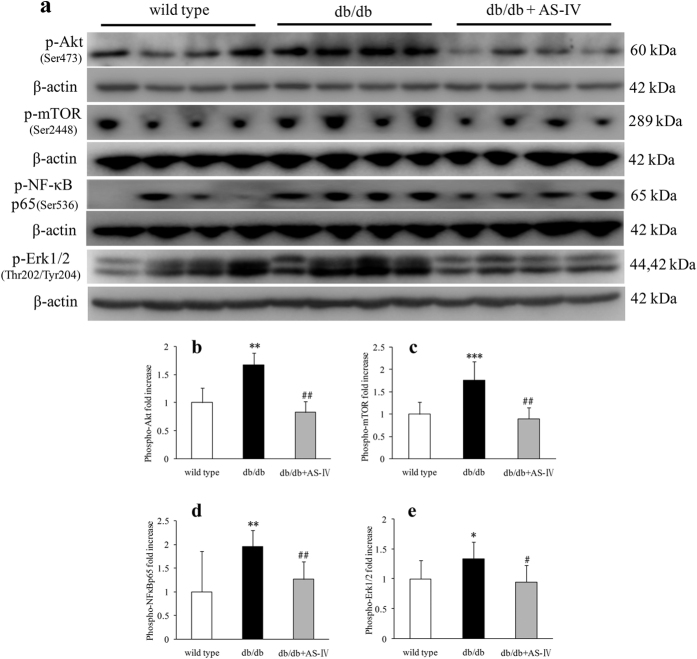
Mice in the db/db group showed more renal cortical expression of p-Akt(Ser473), p-mTOR(Ser2448), p-NF-κB p65(Ser536) and p-Erk1/2(Thr202/Tyr204) than wild type mice, as indicated by Western blot respectively. AS-IV treatment suppressed activation of these proteins (Fig. 7). n = 4–9 per group. *P < 0.05, **P < 0.01 and ***P < 0.001 relative to the wild type group. ^#^*P* < 0.05 and ^##^*P* < 0.01 relative to the db/db group.

Mice in db/db group displayed higher renal cortical expression of p-Akt(Ser473), p-mTOR(Ser2448), p-NF-κB p65(Ser536) and p-Erk1/2 (Thy202/Tyr204) than in wild type mice, as indicated by western blot analysis. AS-IV treatment suppressed the activation of these proteins (Fig. 7).

### AS-IV does not show hepatotoxicity

**Figure 8:**
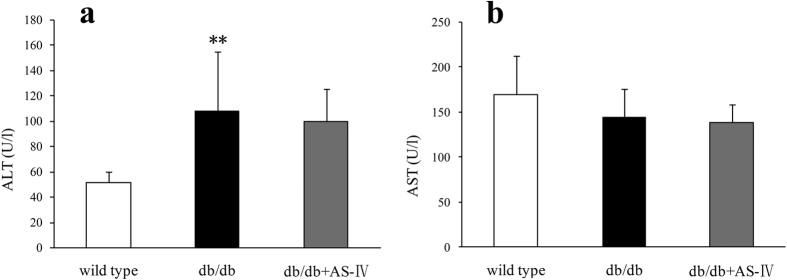
Compared to wild type mice, the db/db mice showed visibly higher ALT and slightly but not significantly lower AST (Fig. 8a,b). There were no significant differences in ALT and AST between the db/db and AS-IV treatment groups (Fig. 8a,b). n = 8 per group. **P < 0.01 relative to the wild type group.

At 12 weeks, db/db mice showed obviously higher alanine transaminase (ALT) than in wild type mice and slightly but not significantly decreased aspartate transaminase (AST) levels (Fig. 8a,b). There were no significant differences in ALT or AST between the db/db and AS-IV treatment groups (Fig. 8a,b).

## Discussion

The results of the present study demonstrated that AS-IV is beneficial for type 2 DN, which might be associated with inhibition of Akt and its associated mTOR, NFκB and Erk1/2 signaling pathway.

The main finding of this experiment was that AS-IV reduced the level of albuminuria in db/db mice. The level of fasting blood glucose and HbA_1C_ was significantly higher in db/db mice than in controls, but AS-IV treatment did not exhibit any obviously influences on these factors. Some studies have demonstrated that AS-IV lowers blood glucose levels in streptozotocin (STZ)-induced diabetic models^25^,^26^. However, AS-IV was found to have no hypoglycemic effect on STZ-induced diabetic rats in other experiments^24^,^27^. The reasons for these conflicting results might involve the dose of AS-IV, the treatment period, and the different model of diabetes used^28^. Along with these findings, the reduction of albuminuria level in AS-IV treated db/db mice may not depend on the hypoglycemic effects. It is implicated that AS-IV directly alleviated glomerular and tubular injury in type 2 diabetes model.

Diabetic individuals show a positive correlation between the level of albuminuria and each of the structural lesions, including GBM thickness, mesangial expansion, and FPW^29^,^30^. Results indicated that AS-IV treatment in db/db mice attenuated glomerular injury, including enlarged glomerular tuft, thickened GBM, and wider FPW, which might contribute to the decreased albuminuria in AS-IV treated db/db mice. Recent study has show that AS-IV ameliorated high glucose-induced podocyte adhesion dysfunction through regulation of α3β1 integrin and integrin-linked kinase^31^. Although there was visibly more deposition on the extracellular matrix in db/db mice, AS-IV did not reduce the mesangial matrix fraction. As one of the main components in the mesangium, fibronectin was also not visibly decreased by AS-IV, which was consistent with the changes observed in the mesangial matrix fraction in each group of mice. The glomerulus in diabetic mice fed AS-IV was visibly smaller than in diabetic mice, and the mesangial volume fraction was no different from that of diabetic mice. Results indicated that the absolute mesangial volume was lower in diabetic mice treated with AS-IV than in diabetic mice. AS-IV treatment not only alleviated glomerular lesions, but also tubular injury in db/db mice. The biomarkers of tubular injury, NAG, NGAL and TGF-β1 were all reduced by AS-IV treatment. Consistent with these results, AS-IV reversed the increase in the proximal tubular area, tubular lumen area, tubular wall area, and thickened TBM in db/db mice. The amelioration of these “loose structures” of the glomeruli and tubules might be the morphological aspect of AS-IV treatment.

Ccr was used to represent the glomerular filtration rate in this experiment. Although AS-IV therapy reduced the glomerular tuft area, it did not prevent hyperfiltration in db/db mice. Hemodynamic, vasoactive, tubular, growth-promoting, and metabolic factors all contribute to diabetic hyperfiltration^32^. Enlarged glomeruli are a pathological feature of early DN, and they can increase the total surface area for filtration. In the current experiment, the area of glomerular tuft was significantly greater in db/db mice. Increased mesangial volume fraction was also observed in db/db mice, which was compensated by glomerular enlargement. Although the area of glomerular tuft was visibly decreased, the glomerular filtration rate did not change in AS-IV treated mice. One reason may account for this phenomenon. AS-IV treated mice showed no decrease in mesangial expansion. This showed a strong inverse correlation with filtration surface^29^.

The mechanisms underlying the protection of AS-IV on diabetic kidney might be complicated. The Akt/mTOR, NFκB, and Erk1/2 signaling pathways play an important role in DN^33^,^34^,^35^. One previous study demonstrated that Gas6 induced mesangial hypertrophy in DN via Akt/mTOR pathway^36^. Akt/mTOR was also involved in high-glucose-and high-insulin-induced injury to renal proximal tubular epithelial cells through GSK3β and eIF2Bε^37^,^38^. Overexpression of connexin43 has been found to reverse high glucose-induced hypertrophy of mesangial cells through regulation of PTEN/Akt/mTOR signaling^39^. Rapamycin, an inhibitor of mTOR, was found to reduce albuminuria, glomerular enlargement, and GBM thickening through down-regulation the enhanced levels of renal phospho-Akt, phospho-p70S6 kinase, and phospho-ribosomal S6 protein in STZ-induced diabetic rats^10^,^11^. More importantly, a recent *in vitro* experiment demonstrated that AS-IV reduced high glucose-induced mesangial damage via Akt/NFκB pathway^21^. These data indicated that Akt and downstream proteins were activated in diabetic kidney and inhibition of these proteins might benefit the treatment of DN.

In conclusion, evidence is here provided that AS-IV, a single-monomer constituent of the Chinese traditional medicine Radix Astragali, can reduce albuminuria and ameliorate glomerular and tubular injury in a mouse model of type 2 diabetes without hepatotoxicity. The mechanisms underlying the protection of AS-IV on DN might be complicated by its inhibition of Akt/mTOR, NFκB and Erk1/2 signals.

## Methods

### Animal model

Eight week-old male db/db mice (BKS.Cg-Dock7^m^+/+Lepr^db^/JNju) and lean wild type littermates were purchased from the Model Animal Research Center of Nanjing University. Animal studies were performed in accordance with relevant guidelines and regulations and approved by the Guangzhou University of Chinese Medicine Institutional Animal Care and Use Committee. Animals were housed at constant room temperature (20 ± 1 °C) under a controlled 12 h light to 12 h dark cycle and had free access to water and food. The experimental mice were randomly allocated to the following groups (n = 8–10 per group): wild type mice fed regular chow, db/db mice fed regular chow, and db/db mice fed a diet supplemented with AS-IV (db/db+AS-IV group). AS-IV purchased from ChengDu ConBon Biotech Co., LTD (China) was added to the standard chow at 1 g/kg diet. The treatment period lasted 12 weeks.

### Physiological and metabolic parameters

Every 2 weeks, the blood glucose was measured using a blood glucose meter (Roche, Basel, Switzerland); the urine was collected using metabolic cages (Tecniplast S.p.a, Buguggiate, Italy); and the body weight was measured. After 12 weeks treatment, the mice were sacrificed and blood samples and kidney tissues were collected. HbA_1C_ was measured using an Ultra2 HbA_1C_ Analyzer. Urine and serum biochemical indexes (urine creatinine, glucose, NAG, serum creatinine, ALT, AST) were detected using a Roche automatic biochemical analyzer. Ccr was calculated using urinary creatinine × urine volume×1000/serum creatinine/1440, and was expressed as microliters per minute.

### Tissue preparation

Immediately after the mice were sacrificed, the kidneys were dissected, weighed, and rinsed in phosphate buffer solution. Then the 10% formalin-fixed kidney was used for histopathological examination and immunohistochemical studies. Sections of renal cortex 1 mm^3^ in volume were fixed in 2.5% glutaraldehyde followed by postfixation in 1% osmic acid for the assays of electromicroscopy. The remaining renal tissues were immediately snap-frozen in liquid nitrogen and stored at −80 °C for later analysis.

### Light microscopy

Paraffin sections (4 μm thick) were stained with PAS to evaluate glomerular and tubular alteration. In each section, 40–50 renal GTA, 20–30 renal glomerular mesangial matrix area, 80–100 renal tubular area and tubular lumen area (axial ratio less than 1.5) were measured using NIS-Elements imaging software Version 4.10 (Nikon Corporation, Tokyo, Japan). The renal GTV was calculated using the method developed by Weibel^40^. This method requires only determination of the mean glomerular random cross-sectional area and calculated using the following formula:V_G_ = Area^1.5^ × β/K, where V_G_ means glomerular volume, β = 1.38 pertains to spheres, and K (a distribution coefficient) was set at 1.10. The tubular wall area was calculated by subtracting renal tubular area from tubular lumen area.

### Electron microscopy

Image J software was used to analyze images collected by EM (JEM-1400, JEOL Ltd., Tokyo, Japan) under×12,000 magnification. GBM and TBM (10–13 photographs in each sample, n = 3 per group) were measured using the grid intersect method^41^. The average podocyte FPW = (π/4) × (∑GBM length/∑number of foot process)^42^ was measured; and the FPW (6 photographs in each sample, n = 3 per group) was presented as nanometer (nm).

### Immunohistochemistry

In brief, kidney paraffin sections (4 μm thick) were mounted on slides, dewaxed and rehydrated. Slides were brought to the boil in 10 mM sodium citrate buffer (pH 6) for 20 min and cooled for 30 min to RT. After 3% hydrogen peroxide treatment for 10 min, the sections were blocked with goat serum for 30 min, followed by the incubation of rabbit polyclonal primary antibody to fibronectin (ab2413, 1:200, Abcam, Cambridge, U.K.) overnight at 4 °C. The sections were then washed with rinse buffer, and incubated with HRP-Polymer Conjugated anti-Mouse/Rabbit IgG complex (Maixin-Bio, Fuzhou, China) for 15 min at room temperature. Localization of peroxidase conjugates was determined using diaminobenzidine tetrahydrochloride solution as chromogen and counterstained with hematoxylin.

### ELISA

Serum insulin (Merck, Darmstadt, Germany), urine albumin (Bethyl Laboratories, Montgomery, TX, U.S.), TGF-β1 (DAKEWE, Shenzhen, China) and NGAL (R&D Systems, Minneapolis, MN, U.S.) were detected by ELISA according to manufacturer’s instructions.

### Western blot

Snap-frozen kidney tissues were homogenized in lysis buffer as described previously. Lysate proteins were separated on a 10% SDS-PAGE gel and then transferred to a PVDF membrane (Bio-Rad Laboratories, Hercules, CA, U.S.). The membrane’s nonspecific binding sites were blocked at room temperature for 1 h with 0.5 g/l non-fat milk powder in Tris-buffered saline/Tween-20 (TBST) and then incubated overnight at 4 °C with primary antibodies. After washed with TBST, the membranes were incubated with secondary antibodies for 1 h at room temperature with shaking. After washing, protein bands were detected and analyzed using a ChemiDoc™ MP Imaging System (Bio-Rad Laboratories, CA, U.S.). β-actin was used as a loading control. Results were expressed as the integrated optical density relative to β-actin. P-Akt(#4060) antibody, p-mTOR(#5536) antibody, p-NF-κB p65(#3033) antibody and p-Erk1/2(#4370) antibody were from Cell Signaling Technologies (Danvers, MA, U.S.). β-actin antibody (A2228) was from Sigma Aldrich (St. Louis, MI, U.S.) and fibronectin (ab2413) antibody was from Abcam (Cambridge, U.K.).

### Statistical analysis

Data are expressed as mean ± SD. Statistical differences between two groups were analyzed using the unpaired student’s *t* test. Differences between multiple groups were analyzed using one-way ANOVA. Statistical analyses were performed using SPSS statistical software, version 16.0. *P* < 0.05 was considered statistically significant.

## Additional Information

**How to cite this article**: Sun, H. *et al*. Astragaloside IV ameliorates renal injury in db/db mice. *Sci. Rep.* **6**, 32545; doi: 10.1038/srep32545 (2016).

## Figures and Tables

**Table 1:** Metabolic characteristics of experimental mice (n = 8 per group).

Weeks	Group	Water consumption(ml)	Urine output(ml)	Feces production(g)
0	wild type	2.1 ± 0.5	0.7 ± 0.1	1.1 ± 0.3
	db/db	3.4 ± 0.9**	2.4 ± 0.6***	1.3 ± 0.2
	db/db + AS-IV	3.6 ± 1.1	2.2 ± 1.3	1.3 ± 0.4
8	wild type	3.2 ± 0.7	1.5 ± 0.6	1.0 ± 0.0
	db/db	6.4 ± 1.9***	6.3 ± 1.9***	1.6 ± 0.4***
	db/db + AS-IV	3.5 ± 1.6##	3.3 ± 1.7##	1.1 ± 0.3##
12	wild type	2.4 ± 0.4	1.1 ± 0.1	1.0 ± 0.4
	db/db	6.9 ± 2.0***	5.9 ± 1.6***	1.4 ± 0.3*
	db/db + AS-IV	4.9 ± 1.9	4.0 ± 1.8#	1.0 ± 0.2##

*P < 0.05, **P < 0.01, ***P < 0.001 vs wild type mice. ^#^*P* < 0.05, ^##^*P* < 0.01 vs db/db mice.
